# Ghana’s herbal medicine industry: prospects, challenges and ways forward from a developing country perspective

**DOI:** 10.3389/fphar.2023.1267398

**Published:** 2023-10-03

**Authors:** Alex Asase

**Affiliations:** Centre for Plant Medicine Research, Mampong-Akuapem, Ghana

**Keywords:** clinical trials, Ghana, herbal medicine, herbal pharmacovigilance, health policy, standardization: technology

## Abstract

The herbal medicine industry is one of the fastest growing industries in the world. However, no detailed assessments have been undertaken on how to achieve the benefits of this industry for developing countries. This study examined the herbal medicine industry in Ghana, with a particular focus on its prospects, challenges and ways forward. The prospects of the medicinal plant trade are huge, and include reducing the national health budget, being a source of foreign and domestic income, as well as creation of employment and poverty reduction. However, the industry is currently inundated with several challenges, such as registration of herbal medicine products and practitioners, a lack of clinical trials for herbal products, standards and quality control issues, shortage of raw plant materials for production, and insufficient scientific research to support traditional claims on the pharmacological effects of medicinal plants. I propose a number of interventions to address these challenges: increased government support, capacity building initiatives, improved regulation of herbal medicines, application of modern technology in the manufacturing of herbal products, large-scale cultivation of medicinal plants, and improved packaging and branding for herbal medicines. Both the national government and the private sector have crucial roles to deliver in the development of the herbal medicine industry in a country like Ghana.

## 1 Introduction

Traditional medicine is defined as the sum total of knowledge, skills, and practices based on theories, beliefs, and experiences indigenous to different cultures, whether explainable or not, used in the maintenance of health as well as prevention, diagnosis, improvement, and treatment of physical and mental illnesses (World Health Organization, 2008). Traditional medicine includes the use of plants, animals, fungi or other components of nature (minerals, rocks etc.). It is undeniable that traditional medicine still plays a pivotal role in the primary healthcare in all countries. The World Health Organization (WHO) estimates is that about 80% of the people in the developing world depend on traditional medicine for meeting their primary healthcare needs (World Health Organization, 2008).

Herbal medicine, which comprises crude plant materials, herbal preparations, and finished products that contain parts of plants as active ingredients (Sen et al., 2010; World Health Organization, 2013; Pan, et al., 2014), is the most popular and lucrative forms of traditional medicine. Indeed, it is the oldest and most widely practiced system of medicine in the world. About 35%–40% of the 252 essential medicines considered by WHO for treatment of and management of diseases are derived exclusively from plant origin (World Health Organization, 2013). Unlike the majority of conventional drugs, herbal medicines are usually a complex combination of natural chemical compounds.

The herbal medicine industry is one of the fastest growing industries in the world, due to factors such as consumer preference for natural products, the perception that natural products are effective without side effects, rising costs for synthetic pharmaceutical drugs and budget cuts for modern healthcare (Jibril et al., 2019; Bareetseng, 2022). The global herbal medicinal products market is expected to be valued at USD 177.65 billion by 2029 (https://www.databridgemarketresearch.com). The industry is indisputably a lucrative business in the world market today: in Asian countries such as China, India, Singapore, and Malaysia a significant portion of their total economic revenue is generated from this industry (Jibril et al., 2019). Despite the enormous prospects of the herbal medicine industry for developing countries, especially in Africa, to the best of knowledge, no work has been done to assess the potential and challenges of this industry in Ghana.

This study examined the Ghanaian herbal medicine industry with a particular focus on prospects, challenges and ways forward. It addresses an important gap in knowledge: the lack of comprehensive information concerning the herbal medicine industry, its potential role in the country’s health policy and sustainable development strategies. This paper begins with a background on the development of traditional medicine in Ghana, then considers the prospects and challenges of the herbal medicine industry and ends with suggestions to improve this industry and increase the benefits from it.

## 2 Development of traditional medicine in Ghana

Healthcare in Ghana is pluralistic, and includes the use of biomedical and traditional healing systems for the treatment of illnesses. Biomedicine was introduced in Ghana during the colonial era and brought with it western notions of illness and healing (Twumasi, 1975; Kpobi and Swartz, 2019). The colonial era also brought Christianity in Ghana through whose missions many traditional and cultural practices were prohibited as they were considered primitive and/or demonic (Kpobi and Swartz, 2019). In fact, traditional healing practices were banned outright under the *Native Customs Regulation Ordinance* of 1878 (Senah, 2001). With the attainment of political independence from colonial rule in 1957 efforts have been made to recognize and promote the work of traditional healers in Ghana (Evans-Anfom, 1986; Kpobi and Swartz, 2019).

**FIGURE 1:**
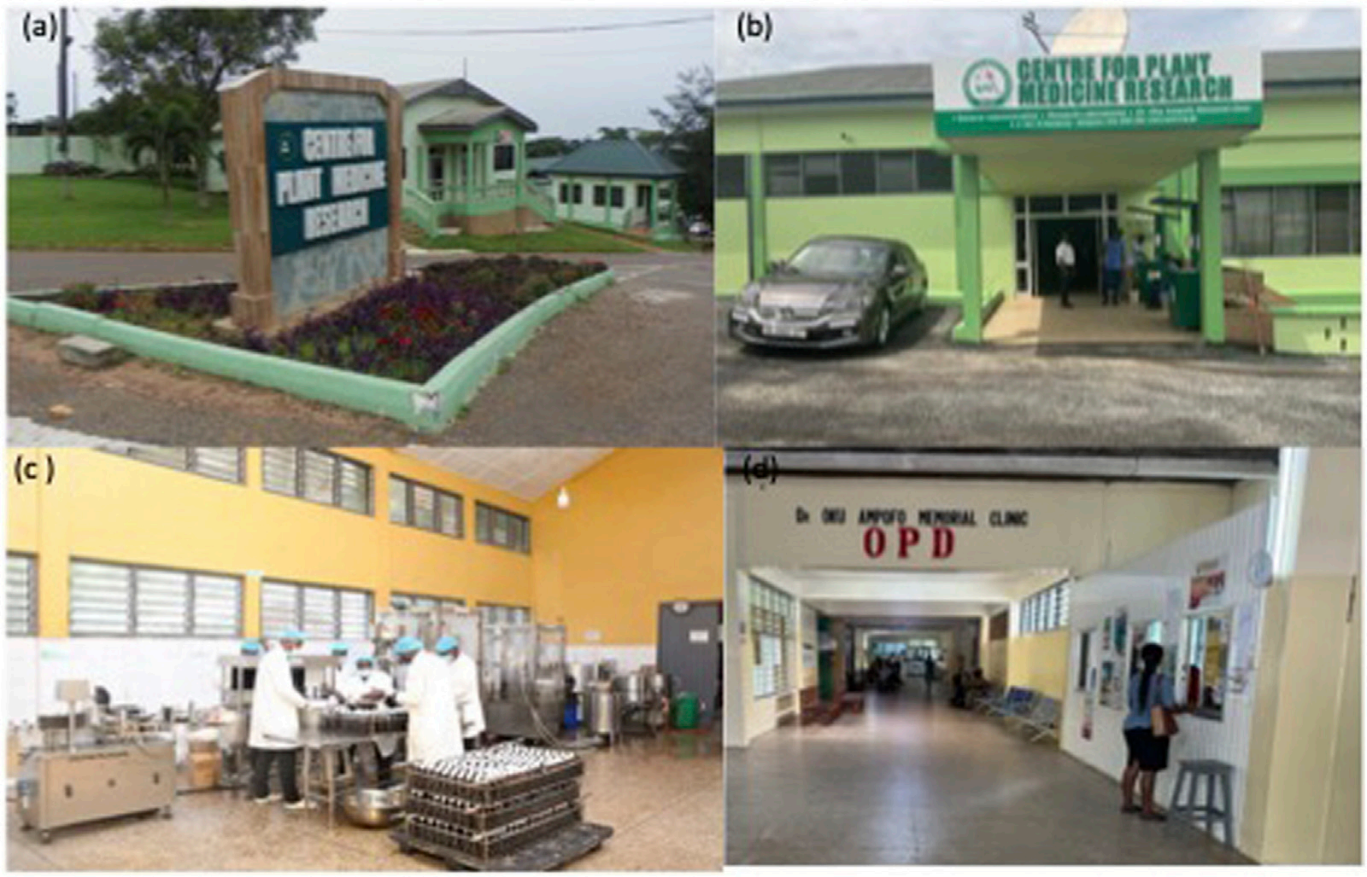
The Centre for Plant Medicine Reserach at Mampong-Akuapem showing **(A)** main entrance to the Centre; **(B)** car part; **(C)** inside of the pradocution facility; and **(D)** Dr. Oku Ampofo Clinic.

Over the years, the Government of Ghana has, supported the development of traditional medicine via the establishment of institutions and the enactment of relevant policies. The Ghana Psychic and Traditional Healers Association was formed in 1961. In 1975, the Centre for Plant Medicine Research (CPMR; formally, the Centre for Scientific Research into Plant Medicine) was established to conduct scientific research into plant medicines (Figure 1). The Traditional and Alternative Medicine Directorate (TAMD) was established in 1999 by upgrading the existing Traditional Medicine Unit (formed in 1991) under the Ministry of Health to oversee policy-related issues. In 2000, the government enacted an act (Act 575) for the establishment of a Traditional Medicine Practice Council (TMPC) with the responsibility for the registration and regulation of all Traditional Medical Practitioners in the country. The Food and Drugs Authority - Ghana (FDA) which was established by the Food and Drugs Law 1992 (PNDC 3058) to control the manufacture, exportation, importation, distribution and licensing of all food and drugs to be marketed have a herbal unit for handling herbal medicines.

In 2012, the government approved the integration of traditional medicine into the healthcare system of Ghana. A year later (2013), the WHO developed and launched the WHO Traditional Medicine Strategy 2014–2023, which also emphasised the integration of traditional and complementary medicine to promote universal healthcare and to ensure the quality, safety and efficiency of such medicines (World Health Organization, 2013). Currently, 55 government hospitals across Ghana have herbal medicine units. Substantial progress has also been achieved in other areas, including the publication of the *Recommended Herbal Medicinal List* (two editions), as well as a publication on basic procedures for assessing safety and efficacy of herbal medicine products (MoH, 2021a). A monograph on medicinal plants of Ghana published by TAMD (Busia, 2009) is currently being updated for a second edition.

Progress has also been achieved in training critical manpower for the herbal medicine industry in Ghana. In 2001, the Department of Herbal Medicine at Kwame Nkrumah University of Science and Technology (KNUST) was established to offer a 4-year BSc Programme in Herbal Medicine. The aim of this department is to train herbal medicine practitioners and manufacturers in application of basic principles, tools, and guidelines; it has trained 241 medical herbalists since its inception. The University of Allied Health Sciences (UHAS) established an Institute of Traditional and Alternative Medicine (ITAM) in 2019, with the mandate to develop and promote all traditional complementary and alternative medicine practices through research, education, and advocacy. The Departments of Pharmacognosy and Herbal Medicine at the University of Cape Coast (UCC) and the University of Ghana (UG) also play crucial roles in capacity building in the field of traditional medicine.

## 3 Prospects for the herbal medicine industry in Ghana

### 3.1 Reduce the national health burden

About 60%–70% of the Ghanaian population depends on traditional medicine (largely herbal medicine) for their primary healthcare needs in the management of both communicable and noncommunicable diseases (Mintah et al., 2022). The ratio of traditional health practitioners to the Ghanaian population was about 1:200, whereas the ratio of medical doctors to population is 1:20,000 (Busia, 2007). Despite substantial progress made over the years, the doctor to population ratio in 2020 for Ghana is 1:6355, which is below the WHO and Commonwealth standard of 1:1,320 and 1:5,000, respectively (NDPC, 2021). The high dependence of Ghanaians on herbal medicine is due to factors such as high accessibility, affordability, and trust (Asase et al., 2008). Herbal medicine has huge prospects of providing solutions in the management and treatment of diseases such as cancer and diabetes, where conventional medicine has so far not provided effective treatment (Houghton, 2009; Andrade-Cetto, 2015). During the recent COVID-19 pandemic, herbal medicines were an important source of hope for most people in Ghana, and elsewhere in the world such as China (Ministry of Science and Technology, 2002). The *Medium-Term National Development Policy Framework (2022–2025)* document has identified traditional medicine as a viable complement to the health system in Ghana (NDPC, 2021).

### 3.2 Economic opportunities

The herbal medicine industry is a major source of employment in the country, as a large number of people such as traditional medicine practitioners, herbal manufacturers, local vendors, raw plant materials aggregators and suppliers, and farmers are involved in the value chain of the herbal medicine industry (Boakye-Yiadom et al., 2023a). The Ghana Federation of Traditional and Alternative Medicine (GHAFTRAM) is currently made up of about 40,000 traditional medicine practitioners, including all practice groups, although 25,000 practitioners are currently registered with the federation (https://ghaftram.org/about-us/). For most of these people, traditional medicine is their main source of livelihood.

The herbal medicine industry has huge prospects for youth employment and poverty reduction in the country. The WHO has projected that the global market for herbal products will be worth US$5 trillion by the year 2050 (Singh et al., 2014). An estimated 951 tons of crude herbal medicines were sold at Ghana’s herbal markets in 2010, with a total value of around US$7.8 million (van Andel et al., 2012). The African Continental Free Trade Area (AfCTA), with its headquarters in Ghana, is a major platform that could be utilized to promote herbal medicine products from Ghana (Boakye-Yiadom et al., 2023b).

It is important to state that herbal medicine is part of Ghana’s national heritage; as such, it could be promoted for purposes of medical tourism, to generate income for the country. Herbal medicine treatment constitutes an important aspect of the health delivery systems in Africa and give identity to a nation through its brand and potency (Houghton, 2009 as cited in Jibril et al., 2019). Herbal medicine should, however be linked to the sustainable use and conservation of biodiversity, as the livelihoods and health of many Ghanaians depend on it (Ministry of Enviornment, Science and Technology (MEST), 2014).

## 4 Challenges of herbal medicine industry in Ghana

### 4.1 Registration of herbal medicines

Despite its enormous benefits and opportunities, the herbal medicine industry in Ghana faces several challenges. A major challenge is the registration of herbal products. For the registration of herbal medicines, the Food and Drugs Authority—Ghana (FDA) requires pharmacological data (efficacy), toxicity data (safety), and clinical data on the herbal medicine. While a large number of herbal products satisfy this requirement for registration, a substantial number of products still fail to be registered, mostly due to microbial contaminants (Mintah et al., 2022). About 50% of herbal products out of 615 products analyzed in 2021 at the CPMR was below quality control standards. Although some training on Good Manufacturing Practices (GMP) has been provided to herbal medicine practitioners and manufacturers, it has not been very intensive. According to Okaiyeto and Oguntibeju (2021), microbial contamination is common in herbal medicines, and it is always difficult for manufactures to prevent or control this contamination.

Another challenge regarding the registration of herbal medicines is the lack of data on shelf life of herbal products. Since almost all herbal medicines for FDA registration do not have data on stability studies, the current practice is to license liquid formulations for only 1 year. However, this interim measure is a huge burden to both the regulator and manufacturers, because of the annual renewal of registration of products. Registration of herbal medicine products is also hampered by unclear guidelines regarding nomenclature of products. Stakeholder consultation is required to bring coherence in the requirements for naming of herbal products for registration. The importance of a standardized nomenclature of pharmacovigilance of herbal medicine has been recommended (Farah et al., 2006).

### 4.2 Certification of herbal medicine practitioners

Certification of traditional medicine practitioners (including herbalists) and their premises in Ghana is done by the Traditional Medicine Practice Council (TMPC). The Traditional medicine Practice Act 575 (2000) provides for the registration of practitioners and qualification for registration. Nevertheless, the certification process is currently fraught with challenges such as inadequate staff, cumbersome procedures, high fees as well as lack of adequate education. As such, there are unscrupulous people who might have taken advantage of unsuspecting practitioners extorting huge sums of monies from them. This also means there could be charlatans operating as licensed partitioners. This unfortunate situation is certainly a source of great worry to TMPC and the general public.

### 4.3 Standardization and quality control

Standardization and quality control are a challenge in the herbal medicine industry, not limited to Ghana, but criticized as a major weakness of herbal medicine worldwide (Srirama et al., 2017). As such, there is usually batch-to-batch variation in the same product. This means patients might not have the same benefits from the use of the same product. Quality control also concerns the harvesting and processing of raw plant materials, detecting possible misidentification of species and adulteration through either substitution of different plant materials or plant parts. Species adulteration in the raw herbal trade market can be as high as 80% in some African countries, due to lack of proper identification methods (Srirama et al., 2017). “Omic” science (genomics, proteomics and metabolomics) is the most recent technology being used in the identification of species adulteration (Pandey et al., 2016), but is rather costly and requires high-end laboratory equipment. Besides, there are instances where manufacturers have added conventional medicines to herbal products for the treatment of specific medical indications (Mintah et al., 2022). It is essential to authenticate and standardize herbal medicines in order to define their efficacy and quality standards to enable clinical trial studies based on phytochemically characterized herbal medicine products to be undertaken (Howes and Simmonds, 2015). The WHO guidelines state that the responsibility of quality assurance of herbal medicinal products has to be shared equally by manufacturers and regulatory bodies (World Health Organization, 1993).

Heavy metal contamination in herbal medicines is a cause of worry and a global threat to human health (Awodele et al., 2013). The potential risk of heavy metal contamination in herbal products due to uptake and accumulation can be a significant source of toxic elements (Okem et al., 2014). The heavy metal content of medicinal plants from some geographical locations in Ghana have been reported to exceed permissible levels, so it is recommended that these levels should be checked before their use for local and pharmaceutical purposes (Annan et al., 2013). Heavy metals may be introduced into medicinal plant products through contamination with agro-chemicals and/or poor production practices (Street, 2012). However, the widespread illegal small-scale mining (popularly called “galamsey’’) activities in the country could be a significant threat to the quality of herbal medicines and subsequently could have adverse effects on health of people consuming such medicines.

### 4.4 Lack of clinical trials to confirm safety and efficacy

The safety and efficacy represent a major challenge facing the herbal medicine industry in Ghana. Clinical trials are required for large-scale endorsement and the safe marketing of herbal medicines at international markets. Besides, bioactivity, pharmacodynamic and toxicity results from *in vitro* bioassay and *in vivo* animal studies might be different from efficacy and toxicity in humans owing to physiologic and metabolic differences between human beings and other animals. To conduct clinical trials on herbal medicines, herbal products must be prepared according to Good Manufacturing Practices (GMP) with appropriate standardization and identification of markers to ensure that the batches of the product being evaluated are always the same (Singh et al., 2014; Parveen et al., 2015; Kumadoh et al., 2023).

In Africa, challenges such as resource constraints, social acceptance, medicine supply, lack of trained staff, and logistical issues have affected the number of clinical trials conducted on herbal medicines (Willcox, 2012). In view of these challenges, it is suggested that a simplified system that takes into consideration factors such as statistical power and sample size for clinical trials on herbal medicines to be considered for Ghana. It would be worth considering a health insurance for clinical trials on herbal medicines instead of trial insurance and indemnity, which comes at higher cost. In Korea, most of the clinical trials on herbal medicines were supported by national governments (Lee et al., 2021). This would be recommendable for Ghana as well. There is currently a strong will on the part of many Ghanaian herbal manufacturers to do clinical trials on their products, but this is impeded by the huge cost associated with such trials.

### 4.5 Scientific research and development

Scientific research on health claims by traditional practitioners is very important to rationalize their use of herbal medicines. A number of supporting documents in the scientific literature have validated the efficacy of herbal medicines for the treatment of different human illnesses (Heinrich and Jager, 2015; MoH, 2021b). However, there are still several gaps that need to be addressed. According to Howes and Simmonds (2015), the issue of efficacy of herbal medicines should be addressed robustly by identifying the active constituent(s) and their modes of action, and determine their polyvalent nature while understanding more about their pharmacokinetic and pharmacodynamic properties. Bioassay-guided fractionation techniques and various methods of structural elucidation are used to identify and characterize bioactive agents in herbal medicines. The mechanisms of action of herbal products may be as a result of synergetic effects or additive effects of compounds in the product (Williamson, 2009).

The CPMR is the institution mandated to conduct scientific research into plant medicines in Ghana (Figure 1). It is directly engaged in basic research, clinical research and practice, and production of herbal medicines (Mintah et al., 2022). Despite some achievements made, the CPMR has numerous challenges, such as inadequate research facilities, human resources and financial backup to enable its full mandate. The CPMR currently lacks facilities for advanced studies including hyphenated techniques such as Liquid Chromatography- Mass Spectrometry (LC-MS), Gas Chromatography- Mass Spectrometry (GC-MS) as well as “Omic” techniques that could greatly enhance medicinal plants research in the country. Collaborative research with national and international partners could be a way to address some of these challenges (Asase et al., 2022).

### 4.6 Shortages of raw plant materials

The herbal medicinal industry in Ghana is threatened by shortages of raw plant materials for production. Most of the commonly used medicinal plants are harvested from the wild, with only a few species (e.g., *Cryptolepis sanguinolenta* (Lindl.) Schltr, *Croton membranaceus* Müll. Arg. and *Mondia whitei* (Hook.f) Skeels) currently under cultivation. The scramble for medicinal plants for local trade and utilization has been documented in different parts of Africa for many years (Moyo et al., 2015). Many of the medicinal plants that are harvested from the wild in the country have become threatened or locally extinct due to unsustainable harvesting, agricultural expansion, forest degradation, deforestation, logging, climate change, and more recently “galamsey” activities (Mshana et al., 2000).

Climate change is a serious threat to the herbal medicine industry through its effects on medicinal plants (Asase and Peterson, 2019). The effects of climate change on medicinal plants include phenological changes (Maikhuri et al., 2018; Applequist et al., 2020), changes in the phytochemical content of plants thereby affecting their pharmaceutical properties (Gairola et al., 2010), decline in plant productivity (Applequist et al., 2020) as well as changes in geographic distribution (Asase and Peterson, 2019). Studies to better understand the effects of climate change on Ghanaian medicinal plants and how it would affect livelihoods of people who depends on these plants is important. The policy implications of climate change on the herbal medicine industry in Ghana will therefore include how people obtain, manage or conserve medicinal plants in the country.

The situation regarding shortages of raw medicinal plant materials is so bad that some materials, such as stem bark of *Khaya senegalensis* (Desr.) A. Juss., must now be sourced from other West African countries for production in Ghana (Mr. Tetteh, *pers comm*.). Although some herbal manufacturers, including the CPMR, the Centre of Awareness (COA), Chocho Industries Ltd., and Taabea Company Ltd., have ventured into cultivation of medicinal plants, a more concerted collaborative approach is needed to address this challenge. Increased public education and awareness may help eradicate any prejudices against cultivation of medicinal plants (Moyo et al., 2015).

### 4.7 Biopiracy

The development of detailed Intellectual Property Right (IPR) and Access and Benefit-Sharing (ABS) policies at the national level to protect and harness the knowledge of herbal medicine practitioners and scientists, as detailed in the Nagoya Protocol on Access and Benefit-Sharing (https://www.cbd.int/abs/), is urgently needed. It is important to note that Ghana has ratified the Nagoya protocol and has some mechanism in place (https://absch.cbd.int/en/countries/GH). According to Reyes-Garcia et al. (2021) a transformative change (i.e., a fundamental system-wide reorganization) in the ways biodiversity policies are designed, implemented and enforced from international to national levels, and across sector requires the foregrounding of indigenous peoples and local communities’ rights and agency in biodiversity policy. Although Ghana has guidelines for Intellectual Property Rights Protection Framework for Indigenous Knowledge Related to Health and Medicinal Plant Resources (2008) there is a lack of awareness among practitioners (Essegbey and Awun, 2015). Most herbal practitioners are not aware about benefits to be derived from patenting traditional knowledge products and services in Ghana. The development of *sui generis* to protect traditional medical knowledge and public awareness is urgently needed, especially with the growing commercial and scientific interest in herbal medicine.

## 5 Ways forward

### 5.1 Increase government support

The Government of Ghana has shown great interest regarding development of the herbal medicine industry over the years. However, important policy gaps remain to be addressed to advance the course of the industry. There is also a need to strengthen and resource existing institutions such as the Centre for Plant Medicine Research to deliver on its mandate. The allocation of funds for research into herbal medicine is of importance to advance the cause of the herbal medicine industry (Kasilo et al., 2023). The National Health Insurance Scheme (NHS) currently does not cover herbal medicines, which makes it impossible for patients to access medicines through that framework. Furthermore, there is no known effort to increase coverage or establish herbal government hospitals, like they exist in China (Parveen et al., 2015). In countries such as China, India, and Korea, traditional medicine is accorded the same importance as modern medicine, and is also included in the national health scheme (Parveen et al., 2015). A national policy on Intellectual Property Right (IPR) and Access and Benefit-Sharing (ABS) on traditional medicine in the country is also needed. It is also critically important that the Traditional Medicine Bill currently at Parliament is passed into law to give more recognition and support for the practice (Mintah et al., 2022).

### 5.2 Capacity building initiatives

There is a clear need for capacity building initiatives in the herbal medicine industry in Ghana (Kumadoh et al., 2023). Herbal practitioners and manufacturers need professional training in Good Manufacturing Practices (GMP), how to collect data on clinical history, and in registration of their herbal products. Capacity enhancement is needed to help herbal practitioners practice their trade more professionally. Training at both technical and highest academic levels (PhD and post-doctoral) are required to address pertinent knowledge gaps in the industry. Training for farmers is needed in Good Agricultural and Collection Practices (GACP), and how to incorporate medicinal plants sustainably into their farming systems (e.g., agroforestry systems) and thereby diversify their sources of income from traditional food and cash crops.

### 5.3 Regulation of herbal medicines industry

Regulation of herbal medicines must be given serious attention and support in the country. Pharmacovigilance of herbal medicines is critically important for developing reliable information on the safety of herbal medicines (Shaw et al., 2012). The safety of herbal medicines has become a serious issue for regulatory authorities as adverse effects have been reported (Awodele et al., 2013). Post-market monitoring of herbal medicine products in Ghana for safety is important. To promote safety of herbal medicine use, certified herbal practitioners should also be trained and encouraged to report adverse drug effects and herb-drug interactions (Howes and Simmonds, 2015). Although the FDA-Ghana has an application system called “Med Safety App” not all practitioners know how to use it in reporting adverse effects of medicines and other health products (www.fdaghana.gov.gh). At the international level, published guidelines exist on pharmacovigilance of herbal medicines and on how to address these challenges (World Health Organization, 2002).

### 5.4 Application of modern technology in manufacturing

It is important to increase the efficiency and production of high-quality herbal medicines that meet international standards (Kumadoh et al., 2023). Although there have been some improvements and innovations in the production and packaging of herbal medicines in the country (Essegbey and Awun, 2015) this is still at the infancy. Most herbal medicines in Ghana are produced on a small scale, and as liquids that are often associated with several undesirable characteristics, such as bitter taste, short shelf-life, dosing and control of microbial contamination during manufacturing as well as challenges with patient compliance. Moreover, the bulky nature of such liquid dosage forms makes the product inconvenient for export outside the Ghanaian market.

The adoption of innovative chemical process engineering methods such as modelling and process intensification with green technology in the manufacturing of herbal medicines could contribute to the economic and ecologic future of the industry (Tegtmeier et al., 2023). It is also important to consider the potential benefits of artificial intelligence (AI) in the herbal medicine industry (Wu et al., 2022). Some herbal manufacturing companies such as Centre of Awareness (COA), Taabea Company Ltd., Angel Herbal Industries Ltd. and Phytotec Ltd. have made some strides in the application of technology in the manufacturing of herbal medicines. But generally, there is a need to support acquisition of advanced technology (equipment and technical know-how) for manufacturing of high-quality herbal medicines which includes converting liquids into solid dosage forms such as capsules and tablets.

### 5.5 Improve packaging and branding

Ghana imports herbal medicines from countries such as India, China, United Kingdom and Germany. The importation of pharmaceutical drugs and medical devices (including herbal medicine) is governed by the Food and Drug Law, 1992 (PNDC Law 305) as amended by the Food and Drug (Amendment) Act, 1996 (Act 523) and the Food and Drugs Board Guidelines (https://www.fdaghana.gov.gh). The imported herbal medicines, from companies such as Himalaya Wellness Company, are well packaged with patient leaflets which are essential for prescription by doctors and other healthcare practitioners. In contrast, most Ghanaian herbal medicines are not well packaged, such that, imported herbal medicines are commonly prescribed.

Given the competitive nature of the business environment, packaging and brand equity provide a competitive advantage to a firm to increase and maintain its market share (Oppong and Phiri, 2018). Therefore, most herbal medicines especially those with export potential in Ghana need to be re-packaged in a form that is more convenient and acceptable to the standards of the international market. According to the study of Oppong and Phiri (2018), brand managers in the plant medicine industry need to consider packaging as an important brand-building tool in their marketing strategy in order to enhance brand equity for the over-the-counter pharmaceutical market. The Ghana Export Promotion Authority (GIPA) and Ghana Investment Promotion Centre (GIPC) have critical roles to play in this branding of herbal medicines from Ghana. It might be worthwhile to initially target the diaspora markets by benefiting from the gains of the AfriCaribbean Trade and Investment Forum 2022 (https://africaribbean-trade-investment-forum-2022.b2match.io/).

### 5.6 Large-scale cultivation of medicinal plants

The sustainability of the herbal industry in Ghana depends on continuous availability and supply of good quality raw plant materials. Indeed, conservation of rare, endangered and threatened medicinal plants is a global concern (Rohini, 2020). There is also an urgent need for the application of biotechnological interventions to conservation and multiplication of threatened medicinal plants. The Biotechnology and Nuclear Agriculture Research Institute (BNARI) of the Ghana Atomic Energy Commission (GAEC) and Plant Genetic Resources Research Institute (PGRRI) of the Council for Scientific and Industrial Research (CSIR-PGRRI) have taken some steps in this direction, but more efforts and funding are needed. Promoting out-grower schemes for farmers to cultivate medicinal plants could also create employment and help ensure availability of medicinal plants, which would be of benefit to the industry. Some species of plants can be harvested sustainable, but there is a need to know the harvesting levels to prevent the resource from exhaustion (van Andel et al., 2012). Scientific research on how to harvest sustainably from the wild is important here.

### 5.7 Increase cooperation and private sector investments

Effective partnerships and collaborations between the various stakeholders are needed to advance the herbal medicine industry in Ghana. These collaborations should be between in-country as well as international partners, and could take various forms such as internships, technology transfer, contract production, and training. Although the benefits to be derived from such cooperation are huge (Asase et al., 2022), there is not much cooperation at the moment because of lack of trust.

At the moment, little private sector investment exists in the herbal medicine industry (Boakye-Yiadom et al., 2023b). Support of the national government to promote and facilitate investment in the herbal industry would be most welcome. Companies such as Kasapreko Company Ltd. (KCL), Adonko Bitters Ltd., and other alcoholic bitters-producing companies that depend on medicinal herbs as their raw materials must be tasked by government with investing in cultivation and/or sustainable harvest of medicinal plants. Pharmaceutical production of herbal medicines is a lucrative business that could help generate considerable foreign revenue for the country.

### 5.8 Research and development

Finally, a national research and development policy on herbal medicine for Ghana is urgently required (Boakye-Yiadom et al., 2023b). Such a national agenda would have the benefit of bringing together all the relevant stakeholders such as researchers, regulators, practitioners, and policymakers to consider capacity needs, policy gaps, funding, and other critical needs. Such steps will ultimately lead to a medium-to long-term strategy for development of the herbal industry in Ghana. It will lead to establishment and strengthening of research networks, partnerships and databases which are needed in the herbal medicine industry (Kasilo et al., 2023).

## 6 Conclusion

The herbal medicine industry in Ghana has seen tremendous development over the years, especially in the area of policy direction, strengthening of institutions, and manpower development. Despite the huge prospects, the industry is currently faced with several challenges that need to be addressed for it to realize the full potential of the industry in Ghana. Both the national government and the private sector are crucial to having an effective national research and development policy for the herbal medicine industry. The issues discussed in this paper are not unique to Ghana, but common for most developing countries, especially in Africa. Comparable strategies could be developed to boost the herbal medicine industry in those countries to take its proper place in national development.

## Data Availability

The original contributions presented in the study are included in the article/Supplementary Material, further inquiries can be directed to the corresponding author.
